# Genetic parameters and expected responses to selection for components of feed efficiency in a Duroc pig line

**DOI:** 10.1186/s12711-017-0362-x

**Published:** 2017-12-01

**Authors:** Juan P. Sánchez, Mohamed Ragab, Raquel Quintanilla, Max F. Rothschild, Miriam Piles

**Affiliations:** 1 Genetica i Millora Animal, IRTA, Torre Marimon, Caldes de Montbui, 08140 Barcelona, Spain; 2 Poultry Production Department, Kafr El-Sheikh University, Kafr El-Sheikh, 33516 Egypt; 3 Department of Animal Science, Iowa State University, Ames, IA 50011 USA

## Abstract

**Background:**

Improving feed efficiency (${\text{FE}}$) is a key factor for any pig breeding company. Although this can be achieved by selection on an index of multi-trait best linear unbiased prediction of breeding values with optimal economic weights, considering deviations of feed intake from actual needs (${\text{RFI}}$) should be of value for further research on biological aspects of ${\text{FE}}$. Here, we present a random regression model that extends the classical definition of ${\text{RFI}}$ by including animal-specific needs in the model. Using this model, we explore the genetic determinism of several ${\text{FE}}$ components: use of feed for growth (${\text{WG}}$), use of feed for backfat deposition (${\text{FG}}$), use of feed for maintenance (${\text{MW}}$), and unspecific efficiency in the use of feed (${\text{RFI}}$). Expected response to alternative selection indexes involving different components is also studied.

**Results:**

Based on goodness-of-fit to the available feed intake (${\text{FI}}$) data, the model that assumes individual (genetic and permanent) variation in the use of feed for maintenance, ${\text{WG}}$ and ${\text{FG}}$ showed the best performance. Joint individual variation in feed allocation to maintenance, growth and backfat deposition comprised 37% of the individual variation of ${\text{FI}}$. The estimated heritabilities of ${\text{RFI}}$ using the model that accounts for animal-specific needs and the traditional ${\text{RFI}}$ model were 0.12 and 0.18, respectively. The estimated heritabilities for the regression coefficients were 0.44, 0.39 and 0.55 for ${\text{MW}}$, ${\text{WG}}$ and ${\text{FG}}$, respectively. Estimates of genetic correlations of ${\text{RFI}}$ were positive with amount of feed used for ${\text{WG}}$ and ${\text{FG}}$ but negative for ${\text{MW}}$. Expected response in overall efficiency, reducing ${\text{FI}}$ without altering performance, was 2.5% higher when the model assumed animal-specific needs than when the traditional definition of ${\text{RFI}}$ was considered.

**Conclusions:**

Expected response in overall efficiency, by reducing ${\text{FI}}$ without altering performance, is slightly better with a model that assumes animal-specific needs instead of batch-specific needs to correct ${\text{FI}}$. The relatively small difference between the traditional ${\text{RFI}}$ model and our model is due to random intercepts (unspecific use of feed) accounting for the majority of variability in ${\text{FI}}$. Overall, a model that accounts for animal-specific needs for ${\text{MW}}$, ${\text{WG}}$ and ${\text{FG}}$ is statistically superior and allows for the possibility to act differentially on ${\text{FE}}$ components.

## Background

Efficiency in the use of feed resources of growing animals is, by far, the most relevant factor for economic sustainability of animal farming. This is particularly true in pig breeding, for which feeding accounts for up to 68% of total variable costs [1]. Estimates of economic weights [2, 3] consistently support the key role of traits that are related to feed efficiency (${\text{FE}}$) in the economic balance of pig production. The availability of devices for automatic recording of individual feed intake (${\text{FI}}$) of animals raised in groups [4] has enabled the implementation of different functions of ${\text{FI}}$ as selection criteria to improve ${\text{FE}}$. One of these is residual feed intake (${\text{RFI}}$), i.e. the deviation of an animal’s ${\text{FI}}$ from the amount of feed predicted to be required for that animal’s biological functions such as maintenance, growth and backfat deposition [5]. Selection for ${\text{RFI}}$ has been demonstrated to be effective in improving ${\text{FE}}$ in pigs [6, 7] and other species [8].

Statistical models that allow the biological components of ${\text{FE}}$ traits to be disentangled have been proposed [9, 10] by considering individual variation within the resource allocation pattern of different biological functions such as growth, maintenance, and fat deposition. In this way, animal-specific requirements are considered instead of those that correspond to the average of all animals in a specific contemporary group, as performed in the classical definition of ${\text{RFI}}$, and this is expected to improve the accuracy of the estimated breeding values (EBV) for ${\text{RFI}}$. In addition, these models provide EBV for each component of ${\text{FE}}$, thus allowing selection for a breeding goal with different weights on the different components of ${\text{FE}}$. This strategy would make it possible to not only reduce the amount of food consumed relative to the animals’ requirements but also manage (e.g. reduce or keep constant) feed requirements differently for each biological function.

In pigs, only Martinsen et al. [11] have implemented these models by considering individual variation in feed allocated for protein and fat deposition. These authors found some relevant genetic variability in these components of global efficiency, but feed requirements for body maintenance were not considered. This biological function is generally defined in relation to the metabolic body weight, i.e. live weight raised to a specific exponent [12].

The objective of our study was to assess genetic parameters for biological components of FE, including body maintenance, growth and fat deposition needs, and to analyze expected responses to selection by considering these components in different indexes.

## Methods

### Animals and selection process

Animals used in this study belonged to a Duroc line that was founded in 1991 [13]. In spite of temporal changes in the economic weights, live weight (W) and backfat thickness (BF) at off test (around 180 days of age) are the traits that had the highest weights in the selection index during the breeding trajectory of this population. Additional traits used for selection were intramuscular fat content, number of piglets born alive and number of functional teats. Recently, other traits associated with fat composition have also been included (*e.g.* oleic acid content).

### Data and traits under study

Individual ${\text{FI}}$ and production traits (W and BF) were recorded for 1076 animals from the aforementioned population during the fattening period at the Center of Porcine Evaluation, IRTA (Monells, Girona, Spain) over four experiments that were carried out from 2004. Table 1 shows the data distribution across experiments and batches of fattening. Animals were weighed and measured for BF (PIGLOG 105, SFK-Technology) between 4 and 11 times during the fattening period. Individual ${\text{FI}}$ was recorded using IVOG^®^ feeding stations (Insentec, Markenesse, The Netherlands). This system records ${\text{FI}}$ per meal, from which individual daily ${\text{FI}}$ was computed as the sum of all meals in a day. Validation of the records for ${\text{FI}}$ per meal was performed following the decision tree proposed by Eissen et al. [4]. When a given meal was declared invalid, a missing record for daily ${\text{FI}}$ was assigned to this animal for that particular day. Missing daily ${\text{FI}}$ records at different ages were predicted using an animal-nested three-coefficient Legendre polynomial function. Live body weight (BW) and BF records were interpolated to the first and last day of each week during the control period, using an animal-nested three-coefficient Legendre polynomial function. Finally, only records from 15 to 25 weeks of age for animals housed in pens with seven or more pen mates were kept for the analyses.

**Table 1:** Distribution of pig data on growth performance across trials and batches

Trial		Batch 1	Batch 2	Batch 3	Batch 4
1	Month of birth	Sept-2003	March-2004	Oct-2004	May-2005
	Number of animals	88	94	68	58
	Range of age (days)	96–188	95–167	102–173	82–181
	Mean number of weights/animal	6.5	5.9	5.6	6.4
2	Month of birth	Sept-2006	Oct-2007	June-2008	
	Number of animals	107	97	106	
	Range of age (days)	69–188	85–190	62–174	
	Mean number of weights/animal	6.0	6.0	5.9	
3	Month of birth	May-2011			
	Number of animals	102			
	Range of age (days)	68–186			
	Mean number of weights/animal	4			
4	Month of birth	Jan-2012	June-2012	Dec-2012	
	Number of animals	115	120	121	
	Range of age (days)	72–174	79–165	75–160	
	Mean number of weights/animal	10.8	9.9	8.9	

In our study, the period considered was between 105 and 182 days of age, during which cumulative ${\text{FI}}$ was used to compute within-week averages of daily ${\text{FI}}$. Similarly, within-week average body weight gains (${\text{WG}}$) and average backfat thickness gains (${\text{FG}}$) were computed after interpolating raw data, as previously described. Weekly metabolic body weight (${\text{MW}}$) was computed as ${\text{BW}}^{0.75}$.

### Statistical analysis models

Eight random regression models were fitted to weekly data. The simplest model corresponds to the classic model for ${\text{RFI}}$:

1 $$y_{ijkl} = BA_{jk} + {\text{MW}}_{\text{ij}} \;\beta_{1j} + {\text{WG}}_{\text{ij}} \;\beta_{2j} + {\text{FG}}_{ij} \;\beta_{3j} + b_{l} + {\text{MW}}_{\text{ij}} \; \gamma_{1jk} + {\text{WG}}_{\text{ij}} \; \gamma_{2jk} + {\text{FG}}_{ij} \;\gamma_{3jk} + a_{1i} + p_{1i} + e_{ijkl} ,$$

where $y_{ijkl}$ denotes the ${\text{FI}}$ record during week $j$ of animal $i$, raised in batch $k,$ and placed in pen $l$. In Model 1, ${\text{FI}}$ records are explained by the systematic effects of the combination of batch and week ($BA_{kj}$; 114 levels), in addition to the fixed partial regressions on ${\text{MW}}$, ${\text{WG}}$, and ${\text{FG}}$ nested within week $j$ ($\beta_{1j}$, $\beta_{2j}$ and $\beta_{3j}$, respectively). The random part of the model includes the corresponding pen effect ($b_{l}$), the random regressions of ${\text{FI}}$ on ${\text{MW}}$, ${\text{WG}}$ and ${\text{FG}}$ nested within each level of week*batch ($\gamma_{1jk}$, $\gamma_{2jk}$ and $\gamma_{3jk}$, respectively), the additive genetic effects ($a_{1i}$), and the permanent environmental effects ($p_{1i}$). The term $e_{ijkl}$ is a random homoscedastic residual.

The other seven models included the same systematic and random terms as Model 1, but differed in the animal-specific components as functions of individual ${\text{MW}}$, ${\text{WG}}$ and ${\text{FG}}$ as follows:

2 $$y_{ijkl} = {\text{Model}} 1 + {\text{MW}}_{\text{ij}} a_{2i} + {\text{MW}}_{\text{ij}} p_{2i} + e_{ijkl}$$

3 $$y_{ijkl} = {\text{Model }}1\varvec{ } + {\text{WG}}_{\text{ij}} a_{3i} + {\text{WG}}_{\text{ij}} p_{3i} + e_{ijkl}$$

4 $$y_{ijkl} = {\text{Model}} 1 + {\text{FG}}_{ij} a_{4i} + {\text{FG}}_{ij} p_{4i} + e_{ijkl}$$

5 $$y_{ijkl} = {\text{Model}} 1 + {\text{MW}}_{\text{ij}} a_{2i} + {\text{WG}}_{\text{ij}} a_{3i} + {\text{MW}}_{\text{ij}} p_{2i} + {\text{WG}}_{\text{ij}} p_{3i} + e_{ijkl}$$

6 $$y_{ijkl} = {\text{Model}} 1 + {\text{MW}}_{\text{ij}} a_{2i} + {\text{FG}}_{ij} a_{4i} + {\text{MW}}_{\text{ij}} p_{2i} + {\text{FG}}_{ij} p_{4i} + e_{ijkl}$$

7 $$y_{ijkl} = {\text{Model}} 1 + {\text{WG}}_{\text{ij}} a_{3i} + {\text{FG}}_{ij} a_{4i} + {\text{WG}}_{\text{ij}} p_{3i} + {\text{FG}}_{ij} p_{4i} + e_{ijkl}$$

8 $$y_{ijkl} = {\text{Model}} 1 + {\text{MW}}_{\text{ij}} a_{2i} + {\text{WG}}_{\text{ij}} a_{3i} + {\text{FG}}_{ij} a_{4i} + {\text{MW}}_{\text{ij}} p_{2i} + {\text{WG}}_{\text{ij}} \;p_{3i} + {\text{FG}}_{ij} \;p_{4i} + e_{ijkl} .$$

where $a_{ni}$ and $p_{ni}$ denote, respectively, the animal-specific additive genetic and permanent environmental partial regressions on standardized weekly ${\text{MW}}$ ($a_{2i}$ and $p_{2i}$), ${\text{WG}}$ ($a_{3i}$ and $p_{3i}$) and ${\text{FG}}$ ($a_{4i}$ and $p_{4i}$). In these models, $a_{1i}$ and $p_{1i}$ act as the corresponding intercepts for individual $i$ and should be interpreted as animal additive genetic and permanent environmental effects for the consumption of feed beyond individual needs, given the biological functions included in the model (i.e. ${\text{MW}}$, ${\text{WG}}$, or ${\text{FG}}$, or combinations of some or all of them). The most complete model (Model 8) assumes that, in addition to an overall effect of ${\text{MW}}$, ${\text{WG}}, {\text{and }}$ ${\text{FG}}$ on ${\text{FI}}$ that is common to all animals in the same batch*week, there is an animal-specific effect on feed allocation pattern (i.e. feed allocation for maintenance, growth and fat deposition) accounted for by the variability of the random regression coefficients $a_{2i}$, $p_{2i}$, $a_{3i}$, $p_{3i}$, $a_{4i}$ and $p_{4i}$.

Bayesian analyses were performed to estimate model parameters. The contribution of the data to the posterior density was considered through a normal conditional likelihood [14], the usual procedure when implementing linear models on normally distributed records. Regarding prior assumptions, the vectors ${\mathbf{BA}}$, ${\varvec{\upbeta}}_{1}$, ${\varvec{\upbeta}}_{2}$ and ${\varvec{\upbeta}}_{3}$ were assumed to follow bounded uniform distributions. The vectors of ${\text{FI}}$ partial regressions on ${\text{MW}}$, ${\text{WG}}$ and ${\text{FG}}$ nested within levels of week*batch (${\varvec{\upgamma}}_{1}$, ${\varvec{\upgamma}}_{2}$ and ${\varvec{\upgamma}}_{3}$), pen (${\mathbf{b}}$), additive genetic (${\mathbf{a}}$), permanent environmental (${\mathbf{p}}$), and residual (${\mathbf{e}}$) effects were assumed to be independent of each other and to follow multivariate normal distributions as follows: ${\varvec{\upgamma}}_{1} \sim{\mathbf{N}}\left( {0,{\varvec{\Gamma}}_{1} } \right)$, ${\varvec{\upgamma}}_{2} \sim{\mathbf{N}}\left( {0,{\varvec{\Gamma}}_{2} } \right)$, ${\varvec{\upgamma}}_{3} \sim{\mathbf{N}}\left( {0,{\varvec{\Gamma}}_{3} } \right)$, ${\mathbf{b}} \sim{\mathbf{N}}\left( {0,{\mathbf{B}}} \right)$, ${\mathbf{a}} \sim{\mathbf{N}}\left( {0,{\mathbf{G}}} \right)$, ${\mathbf{p}} \sim{\mathbf{N}}\left( {0,{\mathbf{P}}} \right)$ and ${\mathbf{e}} \sim{\mathbf{N}}\left( {0,{\mathbf{R}}} \right)$, where ${\varvec{\Gamma}}_{1}$, ${\varvec{\Gamma}}_{2} ,$ ${\varvec{\Gamma}}_{3}$, ${\mathbf{B}}$, ${\mathbf{G}}$, ${\mathbf{P}}$ and ${\mathbf{R}}$ are the respective variance–covariance matrices between the levels of each factor. These (co)variance matrices were assumed as follows for the most complex model i.e. Model 8, (for Models 1–7, they were re-dimensioned in order to account only for the terms considered in the model):

$${\varvec{\Gamma}}_{1} = \sigma_{{\upgamma }_{1}}^{2} \times {\mathbf{I}}_{{\upgamma }_{1}} ,\,\,{\varvec{\Gamma}}_{2} = \sigma_{{\upgamma }_{2} }^{2} \times {\mathbf{I}}_{{\upgamma }_{2}} ,\;{\varvec{\Gamma}}_{3} = \sigma_{{\upgamma }_{3} }^{2} \times {\mathbf{I}}_{{\upgamma }_{3}} ,\;{\mathbf{B}} = \sigma_{b}^{2} \times {\mathbf{I}}_{\varvec{b}}$$

$$\begin{aligned} & {\mathbf{G}} = \left[ {\begin{array}{*{20}c} {\sigma_{{a_{1} }}^{2} } & {\sigma_{{a_{1,} a_{2} }}^{{}} } & {\begin{array}{*{20}c} {\sigma_{{a_{1,} a_{3} }}^{{}} } & {\sigma_{{a_{1,} a_{4} }}^{{}} } \\ \end{array} } \\ {\sigma_{{a_{1,} a_{2} }}^{{}} } & {\sigma_{{a_{2} }}^{2} } & {\begin{array}{*{20}c} {\sigma_{{a_{2,} a_{3} }}^{{}} } & {\sigma_{{a_{2,} a_{4} }}^{{}} } \\ \end{array} } \\ {\begin{array}{*{20}c} {\sigma_{{a_{1,} a_{3} }}^{{}} } \\ {\sigma_{{a_{1,} a_{4} }}^{{}} } \\ \end{array} } & {\begin{array}{*{20}c} {\sigma_{{a_{2,} a_{3} }}^{{}} } \\ {\sigma_{{a_{2,} a_{4} }}^{{}} } \\ \end{array} } & {\begin{array}{*{20}c} {\begin{array}{*{20}c} {\sigma_{{a_{3} }}^{2} } & {\sigma_{{a_{3,} a_{4} }}^{{}} } \\ \end{array} } \\ {\begin{array}{*{20}c} {\sigma_{{a_{3,} a_{4} }}^{{}} } & {\sigma_{{a_{4} }}^{2} } \\ \end{array} } \\ \end{array} } \\ \end{array} } \right] \otimes {\mathbf{A}} = {\mathbf{G}}_{0} \otimes {\mathbf{A}}, \\ & {\mathbf{P}} = \left[ {\begin{array}{*{20}c} {\sigma_{{p_{1} }}^{2} } & {\sigma_{{p_{1,} p_{2} }}^{{}} } & {\begin{array}{*{20}c} {\sigma_{{p_{1,} p_{3} }}^{{}} } & {\sigma_{{p_{1,} p_{4} }}^{{}} } \\ \end{array} } \\ {\sigma_{{p_{1,} p_{2} }}^{{}} } & {\sigma_{{p_{2} }}^{2} } & {\begin{array}{*{20}c} {\sigma_{{p_{2,} p_{3} }}^{{}} } & {\sigma_{{p_{2,} p_{4} }}^{{}} } \\ \end{array} } \\ {\begin{array}{*{20}c} {\sigma_{{p_{1,} p_{3} }}^{{}} } \\ {\sigma_{{p_{1,} p_{4} }}^{{}} } \\ \end{array} } & {\begin{array}{*{20}c} {\sigma_{{p_{2,} p_{3} }}^{{}} } \\ {\sigma_{{p_{2,} p_{4} }}^{{}} } \\ \end{array} } & {\begin{array}{*{20}c} {\begin{array}{*{20}c} {\sigma_{{p_{3} }}^{2} } & {\sigma_{{p_{3,} p_{4} }}^{{}} } \\ \end{array} } \\ {\begin{array}{*{20}c} {\sigma_{{p_{3,} p_{4} }}^{{}} } & {\sigma_{{p_{4} }}^{2} } \\ \end{array} } \\ \end{array} } \\ \end{array} } \right] \otimes {\mathbf{I}}_{\varvec{p}} = {\mathbf{P}}_{0} \otimes {\mathbf{I}}_{\varvec{p}} , \\ & {\text{and}}\quad {\mathbf{R}} = \sigma_{e}^{2} \times {\mathbf{I}}_{\varvec{n}} , \\ \end{aligned}$$

where ${\mathbf{A}}$ is the numerator relationship matrix, of dimension equal to the number of animals in the pedigree, while ${\mathbf{I}}_{{\upgamma }_{1}}$, ${\mathbf{I}}_{{\upgamma }_{2}}$, ${\mathbf{I}}_{{\upgamma }_{3}}$, ${\mathbf{I}}_{\varvec{b}}$, ${\mathbf{I}}_{\varvec{p}}$ and ${\mathbf{I}}_{\varvec{n}}$ are identity matrices of the appropriate dimensions: batch*week levels, pens, animals with records and total number of records. The variance components $\sigma_{{\upgamma }_{1}}^{2}$, $\sigma_{{\upgamma }_{2}}^{2}$, $\sigma_{{\upgamma }_{3}}^{2}$, $\sigma_{b}^{2}$, ${\mathbf{G}}_{0}$, ${\mathbf{P}}_{0}$ and $\sigma_{e}^{2}$ were assumed to follow bounded uniform distributions throughout the valid parameter space.

The Bayesian analyses were performed using Markov chain Monte Carlo (MCMC) techniques. Four independent Gibbs sampling chains were run, which all had the same initial values for all variance components but different random seeds. Each chain elapsed for 1 million iterations. The first 100,000 iterations were discarded as the burn-in period and then one in each 100 samples was retained to perform the characterization of the marginal posterior distributions of the parameters of interest. All analyses were conducted using the gibbs2f90 program [15]. The deviance information criterion (DIC; [16]) was used to compare the models by assessing which model yielded a better fit to the available data, with a penalty for model complexity.

### Simulation of the selection process to predict genetic responses

The process to generate data was designed to resemble the structure of a small pig selection nucleus, formed by 120 sows and 30 boars, in which mating among close relatives was avoided. Sows were kept in production for six parities, although stochastic culling was performed in each batch, according to a Weibull survival function. Litter size at birth in each farrowing was sampled from a normal distribution with a mean of 9 and variance of 6. Mortality and culling rates during lactation and growing periods were jointly set to 0.2. For each animal finishing the growing period, ${\text{FI}}$ records were generated according to the following model:

$${\text{FI}}_{ijbk} =\, \mu + b_{b} + {\text{MW}}_{\text{ij}} \beta_{1} + {\text{WG}}_{\text{ij}} \beta_{2} + {\text{FG}}_{ij} \beta_{3} + a_{1,i} + {\text{MW}}_{\text{ij}} a_{2,i} + {\text{WG}}_{\text{ij}} a_{3,i} + {\text{FG}}_{\text{ij}} a_{4,i} + p_{1,i} + {\text{MW}}_{\text{ij}} p_{2,i} + {\text{WG}}_{\text{ij}} p_{3,i} + {\text{FG}}_{\text{ij}} p_{4,i} + e_{ibjk} .$$

In this equation, $\mu$, $\beta_{1}$, $\beta_{2}$ and $\beta_{3}$ were set to 3000, 235, 64 and 33, respectively. These regression coefficients were defined from the estimates of $\beta_{1,j}$, $\beta_{2,j}$ and $\beta_{3,j}$ obtained with Model 1, averaging coefficients across ages. Realizations of the individual vectors of breeding values (${\mathbf{a}}$), permanent environmental effects (${\mathbf{p}}$), pen effects (${\mathbf{b}}$), and residual deviates (${\mathbf{e}}$) were sampled from the appropriate normal distributions defined by variance components equal to those estimated for Model 8. Note that this simulation model is very close to Model 8, although batch structure and age-dependence patterns were ignored. This was expected to yield more accurate EBV than obtained with real data, but was not expected to affect comparisons between selection strategies.

In a parallel and independent simulation process, the joint distribution of weekly standardized ${\text{MW}}$, ${\text{WG}}, {\text{and }}$ ${\text{FG}}$ was generated as follow:

9 $$\left[ {\begin{array}{*{20}c} {{\mathbf{MW}}} \\ {{\mathbf{WG}}} \\ {{\mathbf{FG}}} \\ \end{array} } \right]\sim N\left[ {\begin{array}{*{20}c} {\varvec{\mu}_{{\varvec{MW}}} + {\mathbf{Z}}_{\varvec{b}} \mathbf{b}_{{\varvec{MW}}} + {\mathbf{Z}}_{\varvec{a}} \mathbf{a}_{{\varvec{MW}}} + {\mathbf{Z}}_{\varvec{p}} {\mathbf{p}}_{{\varvec{MW}}} } \\ {\varvec{\mu}_{{\varvec{WG}}} + {\mathbf{Z}}_{\varvec{b}} {\mathbf{b}}_{{\varvec{WG}}} + {\mathbf{Z}}_{\varvec{a}} {\mathbf{a}}_{{\varvec{WG}}} + {\mathbf{Z}}_{\varvec{p}} {\mathbf{p}}_{{\varvec{WG}}} } \\ {\varvec{\mu}_{{\varvec{FG}}} + {\mathbf{Z}}_{\varvec{b}} {\mathbf{b}}_{{\varvec{FG}}} + {\mathbf{Z}}_{\varvec{a}} {\mathbf{a}}_{{\varvec{FG}}} + {\mathbf{Z}}_{\varvec{p}} {\mathbf{p}}_{{\varvec{FG}}} } \\ \end{array} ,\; {\mathbf{R}}_{\varvec{t}} \varvec{ }} \right],$$

where $\varvec{\mu}_{{\varvec{MW}}}$, $\varvec{\mu}_{{\varvec{WG}}}$ and $\varvec{\mu}_{{\varvec{FG}}}$ were set to 0 and the vectors of pen (${\mathbf{b}}$), additive genetic (${\mathbf{a}}$) and permanent environmental (${\mathbf{p}}$) effects were sampled from the following distributions:

$$\left[ {\begin{array}{*{20}c} {{\mathbf{b}}_{{\varvec{MW}}} } \\ {{\mathbf{b}}_{{\varvec{WG}}} } \\ {{\mathbf{b}}_{{\varvec{FG}}} } \\ \end{array} } \right]\sim N\left[ {\begin{array}{*{20}c} 0 \\ 0 \\ 0 \\ \end{array} ,{\mathbf{B}}_{\varvec{t}} } \right];$$

$$\left[ {\begin{array}{*{20}c} {\mathbf{a}_{{\varvec{MW}}} } \\ {\mathbf{a}_{{\varvec{WG}}} } \\ {\mathbf{a}_{{\varvec{FG}}} } \\ \end{array} } \right]\sim N\left[ {\begin{array}{*{20}c} 0 \\ 0 \\ 0 \\ \end{array} ,{\mathbf{G}}_{\varvec{t}} } \right];$$

$$\left[ {\begin{array}{*{20}c} {\mathbf{p}_{{\varvec{MW}}} } \\ {\mathbf{p}_{{\varvec{WG}}} } \\ {\mathbf{p}_{{\varvec{FG}}} } \\ \end{array} } \right]\sim N\left[ {\begin{array}{*{20}c} 0 \\ 0 \\ 0 \\ \end{array} ,{\mathbf{P}}_{\varvec{t}} } \right].$$

As (co)variance values for the matrices ${\mathbf{B}}_{\varvec{t}}$, ${\mathbf{G}}_{\varvec{t}}$ and ${\mathbf{P}}_{\varvec{t}}$, we took the EM-REML estimates that were obtained by fitting a multi-trait model to actual standardized weekly ${\text{MW}}$, ${\text{WG}}, {\text{and }}$ ${\text{FG}}$. In this EM-REML analysis, the combination of batch by week of age was also considered as a fixed factor, in addition to the random effects indicated in Eq. (9). We used the following (co)variance values:

$${\mathbf{B}}_{\varvec{t}} = \left[ {\begin{array}{*{20}c} {0.25} & {0.05} & {0.02} \\ {0.05} & {0.02} & {0.02} \\ {0.02} & {0.02} & {0.02} \\ \end{array} } \right] \otimes {\mathbf{I}}_{\varvec{b}} ,$$

$${\mathbf{G}}_{\varvec{t}} = \left[ {\begin{array}{*{20}c} {0.44} & {0.21} & {0.14} \\ {0.21} & {0.22} & {0.15} \\ {0.14} & {0.15} & {0.20} \\ \end{array} } \right] \otimes {\mathbf{I}}_{\varvec{g}} ,$$

$$\varvec{ }{\mathbf{P}}_{\varvec{t}} = \left[ {\begin{array}{*{20}c} {0.31} & {0.19} & {0.08} \\ {0.19} & {0.21} & {0.07} \\ {0.08} & {0.07} & {0.09} \\ \end{array} } \right] \otimes {\mathbf{I}}_{\varvec{p}} ,$$

$${\mathbf{R}}_{\varvec{t}} = \left[ {\begin{array}{*{20}c} {0.04} & {0.03} & {0.02} \\ {0.03} & {0.53} & {0.17} \\ {0.02} & {0.17} & {0.67} \\ \end{array} } \right] \otimes {\mathbf{I}}_{\varvec{n}} ,$$

Phenotypic records were generated for all offspring from all parities of all sows. However, genetic evaluation at each generation was conducted after the second parity, i.e. considering only animals that were born in the first two parities as selection candidates; animals from later parities contributed phenotypic information that was used for genetic evaluation in the next generation.

For genetic selection, six scenarios were defined according to the selection criterion used to rank candidates ($i$), hereafter denoted as $\hat{I}_{I,i}$ (the subindex $I$ stands for ${\text{FI}}$, ${\text{tRFI}}$, ${\text{RFI}}$, ${\text{FI}}$/${\text{MW}}$, FI/${\text{WG}}$ and ${\text{FI}}$/${\text{FG}}$):

1. Selection against feed intake using the EBV obtained from an animal model on ${\text{FI}}$ that included the same fixed terms as Model 1 except for the partial regressions on biological functions ($\hat{I}_{FI,i} = - \hat{a}_{1,i}$). Our aim here was to explore the consequences of selecting for reduced raw feed intake.
2. Selection to reduce the traditional definition of ${\text{RFI}}$, in which individual needs are exclusively defined by the fixed regression terms included in Model 1 ($\hat{I}_{tRFI,i} = - \hat{a}_{1,i}$ under Model 1).
3. Selection against the consumption of feed beyond individual needs according to Model 8 ($\hat{I}_{RFI,i} = - \hat{a}_{1,i}$ under Model 8). In this case, needs are defined by the overall needs (multiple fixed regressions) plus the specific needs that are individually associated with the animal’s ${\text{MW}}$, ${\text{WG and }}$ ${\text{FG}}$.
4. Selection for reduced feed required per unit of ${\text{MW}}$ according to Model 8 ($\hat{I}_{FI/MW,i} = - \hat{a}_{2,i}$ under Model 8).
5. Selection for reduced feed required per unit of ${\text{WG}}$ according to Model 8 ($\hat{I}_{FI/WG,i} = - \hat{a}_{3,i}$ under Model 8).
6. Selection for reduced feed required p*e*r unit of ${\text{FG}}$ according to Model 8 ($\hat{I}_{FI/FG,i} = - \hat{a}_{4,i}$ under Model 8).

Once the selection candidates were evaluated, the best 120 females were selected from the whole group of female candidates, while for males the best ranked animal within each sire family was selected. This selection procedure was repeated for three generations, and 50 replicates were run for each scenario. Predictions of breeding values for feed intake components were obtained using EM-REML, with variance components estimated for each generation and replicate. Genetic evaluation accuracy was computed as the correlation between actual and predicted genetic values for the different indexes.

## Results

Table 2 presents descriptive statistics of the analysed traits by week of fattening. Means and standard deviations were within the range of values reported in the literature for other pig populations [17–19]. The average feed conversion ratio (${\text{FI}}$/${\text{WG}}$) ranged from 2.6 in the first week to nearly 3.5 during the last 3 weeks of control. The limited efficiency of this Duroc population was the result of the large depth of subcutaneous fat deposits; mean ${\text{BF}}$ ranged from 10 mm at 16 weeks of age up to 22 mm at 26 weeks (results not shown). Fairly constant ${\text{WG}}$ was observed throughout the fattening period, whereas both daily ${\text{FI}}$ and ${\text{FG}}$ increased linearly with age. This pattern was previously reported for other pig populations [20]. Raw correlations of 0.46, 0.25 and 0.42 were estimated between ${\text{MW}}$ and ${\text{BW}}$, ${\text{MW}}$ and ${\text{BF}}$, and ${\text{BW}}$ and ${\text{FG}}$, respectively.

**Table 2:** Mean (standard deviation) of analysed growth phenotypes by age

Age (weeks)	${\mathbf{FI}}$ (kg)	Weight (kg)	${\mathbf{MW}}$ (kg)	${\mathbf{WG}}$ (kg/d)	${\mathbf{FG}}$ (mm/d)	N
15	2.22 (0.41)	50.92 (6.88)	19.85 (1.97)	0.85 (0.12)	0.13 (0.07)	1070
16	2.39 (0.44)	56.85 (7.3)	21.47 (2.05)	0.84 (0.12)	0.13 (0.07)	1069
17	2.51 (0.48)	62.76 (7.79)	23.06 (2.12)	0.85 (0.12)	0.13 (0.07)	1066
18	2.69 (0.51)	68.68 (8.25)	24.62 (2.20)	0.87 (0.13)	0.14 (0.07)	1062
19	2.85 (0.52)	74.80 (8.75)	26.17 (2.26)	0.87 (0.13)	0.14 (0.07)	1058
20	2.91 (0.52)	80.90 (9.10)	27.71 (2.32)	0.88 (0.14)	0.15 (0.07)	1055
21	2.98 (0.54)	86.97 (9.49)	29.21 (2.38)	0.89 (0.14)	0.16 (0.08)	1043
22	3.03 (0.53)	93.52 (9.90)	30.80 (2.42)	0.90 (0.15)	0.16 (0.08)	1013
23	3.10 (0.54)	100.11 (10.46)	32.35 (2.53)	0.89 (0.15)	0.17 (0.08)	906
24	3.04 (0.55)	106.58 (10.85)	33.87 (2.59)	0.89 (0.15)	0.17 (0.09)	732
25	3.07 (0.60)	113.82 (10.53)	35.55 (2.46)	0.91 (0.15)	0.18 (0.09)	516

${\text{FI}}$ = feed intake; Weight = weight at the end of the week; ${\text{MW}}$ = mean metabolic weight of the week; ${\text{WG}}$ = weight gain; ${\text{FG}}$ = backfat thickness gain; N = number of animals

### Model comparisons

Table 3 shows the DIC values across chains for the eight models analysed. DIC results from Models 2, 3 and 4 can be used to qualitatively examine the magnitude of the individual variation in efficiency that is associated with each explanatory variable. The strongest effect, by far, was associated with feed allocated for ${\text{WG}}$ (Model 3), followed by ${\text{MW}}$ (Model 2) and ${\text{FG}}$ (Model 4), for which the differences were smaller.

**Table 3:** Deviance information criterion values for each model and chain run

Model	Chain1	Chain2	Chain3	Chain4	Chain5	Range^a^
Model 8	148,566	148,579	148,561	148,575	148,563	18
Model 5	148,622	148,614	148,617	148,626	148,623	12
Model 7	148,677	148,675	148,672	148,670	148,681	11
Model 6	148,711	148,706	148,711	148,729	148,714	23
Model 3	148,721	148,721	148,724	148,722	148,719	5
Model 2	148,808	148,812	148,807	148,807	148,808	5
Model 4	148,829	148,831	148,830	148,831	148,828	2
Model 1	148,942	148,941	148,941	148,940	148,941	2

^a^Range = range across chains for a given model

Differences in DIC between Models 1 to 4 and Models 5 to 7 were larger than within-model variation across sampling chains. This indicates that Monte Carlo errors did not prevent models that fitted more than one biological component as random regression term (Models 5, 6 and 7) to be declared preferable over those that fitted just one individual component of ${\text{FE}}$ (Models 2, 3 and 4) or over the traditional ${\text{RFI}}$ definition (Model 1). DIC results indicate that Model 8 was the most appropriate, i.e. the model that considers animal-specific variation (genetic and permanent) in the use of feed for maintenance, ${\text{WG}}$ and ${\text{FG}}$, fitted the ${\text{FI}}$ data better than any other model.

### Parameter estimates

Table 4 includes the regression coefficients of ${\text{FI}}$ on ${\text{MW}}$, ${\text{WG and }}$ ${\text{FG}}$ nested by week ($\beta_{1j}$, $\beta_{2j}$ and $\beta_{3j}$), as obtained from Model 1. These regressions must be interpreted as the amount of feed required to support an increase of the corresponding explanatory variable by one standard deviation. Since ${\text{MW}}$, ${\text{WG}}\;{\text{and }}$ ${\text{FG}}$ were considered in standardized units within week*batch class, these coefficients are directly comparable to each other. According to the estimated regression coefficients, ${\text{MW}}$ is the explanatory variable with the largest relative weight on feed consumption in any period, although it declined with age, from 295 (at 15 weeks) to 157 (at 25 weeks) g/d per ${\text{MW}}$ sd unit. An opposite pattern was observed for regression coefficients on ${\text{WG}}$, increasing from 2 (at week 16) to 146 (at week 25) g/d per ${\text{WG}}$ sd unit. Feed requirements per sd unit of ${\text{FG}}$ were the lowest and followed a pattern that could be considered constant over time. The variability of these regression coefficients across levels of week*batch (results not shown) followed the same pattern as the fixed coefficients. The largest variability was observed for ${\text{MW}}$ (posterior mean (posterior sd)): 2329 (531) (g/MW sd units)^2^, then for ${\text{WG}}$: 1166 (344) (g/ ${\text{WG}}$ sd units)^2^, and finally for ${\text{FG}}$: 328 (194) (g/${\text{FG}}$ sd units)^2^.

**Table 4:** Mean (standard deviation) of marginal posterior distributions for within-week regression coefficients of feed intake on standardized^a^ metabolic weight (${\mathbf{MW}}$), weight gain (${\mathbf{WG}}$) and backfat thickness gain (${\mathbf{FG}}$)

Age (week)	Regression coefficients of daily feed intake (g/d) on standardized units of		
	${\mathbf{MW}}$	${\mathbf{WG}}$	${\mathbf{FG}}$
15	295 (19)	13 (16)	16 (11)
16	286 (19)	2 (16)	36 (11)
17	284 (19)	8 (15)	24 (11)
18	266 (19)	13 (15)	37 (11)
19	272 (19)	49 (15)	24 (11)
20	220 (18)	57 (15)	37 (11)
21	210 (18)	96 (15)	38 (11)
22	205 (18)	101 (14)	24 (11)
23	192 (20)	102 (15)	56 (12)
24	193 (21)	120 (17)	23 (13)
25	157 (24)	146 (20)	49 (16)

^a^Standardization was done within level of week by batch combination

Estimates of genetic parameters for Model 8 evaluated at the means of the explanatory covariates (zero) are in Table 5. Descriptive statistics of the marginal posterior distributions were obtained after merging samples from the five independent chains. Therefore, effective sample size was the sum of the corresponding values for each chain. The estimate of heritability (posterior mean (posterior sd)) for ${\text{RFI}}$ from Model 8 was 0.12 (0.05) (Table 5). This parameter was defined as the ratio between the additive genetic variance for the intercept and the sum of additive genetic plus permanent variances for the intercept, plus pen and residual variances; note that variances of random regressions terms are not considered because the estimate is reported at the means of these terms, which were zero. As expected, the above estimated heritability was lower than that estimated for ${\text{RFI}}$ under Model 1 (0.18 (0.06)) (result not shown).

**Table 5:** Statistics^a^ of marginal posterior distributions for genetic parameter estimates under Model 8 at the mean of the explanatory covariates

Genetic parameter^b^	Mean	Median	sd	HPD	Pr| $\hat{\varvec{p}}$ |>0^c^	ESS	
$\hat{h}_{RFI}^{2}$	0.12	0.13	0.05	0.04	0.20	1.00	476
$\hat{h}_{FI/MW}^{2}$	0.44	0.43	0.20	0.10	0.79	1.00	155
$\hat{h}_{FI/WG}^{2}$	0.39	0.41	0.19	0.06	0.68	1.00	301
$\hat{h}_{FI/FG}^{2}$	0.55	0.56	0.14	0.31	0.79	1.00	356
r_g_(${\text{a}}_{\text{RFI}}$, ${\text{a}}_{{{\text{FI}}/{\text{MW}}}}$)	− 0.46	− 0.48	0.30	− 0.99	0.08	0.07	239
r_g_(${\text{a}}_{\text{RFI}}$, ${\text{a}}_{{{\text{FI}}/{\text{WG}}}}$)	0.75	0.82	0.26	0.23	1.00	0.98	116
r_g_(${\text{a}}_{\text{RFI}}$, ${\text{a}}_{{{\text{FI}}/{\text{FG}}}}$)	0.52	0.51	0.31	0.02	0.98	0.96	355
r_g_(${\text{a}}_{{{\text{FI}}/{\text{MW}}}}$, ${\text{a}}_{{{\text{FI}}/{\text{WG}}}}$)	− 0.43	− 0.46	0.33	− 0.96	0.12	0.10	203
r_g_(${\text{a}}_{{{\text{FI}}/{\text{MW}}}}$, ${\text{a}}_{{{\text{FI}}/{\text{FG}}}}$)	− 0.19	− 0.21	0.30	− 0.74	0.41	0.25	326
r_g_(${\text{a}}_{{{\text{FI}}/{\text{WG}}}}$, ${\text{a}}_{{{\text{FI}}/{\text{FG}}}}$)	0.57	0.60	0.19	0.19	0.88	0.99	342

^a^Mean, median, highest posterior density (HPD) intervals, probability of the parameter to be higher than zero (Pr| $\hat{p}$ | > 0) and effective sample size (ESS)

^b^ $\hat{h}_{RFI}^{2}$ = heritability estimate of the intercept (residual feed intake); $\hat{h}_{j}^{2}$ = heritability estimate (defined as the ratio between additive variance associated to each component divided by the sum of their permanent and additive genetic components) of the slopes of ${\text{FI}}$ on the different explanatory variables $j$: metabolic weight (${\text{FI}}$ / ${\text{MW}}$), overall growth (${\text{FI}}$ / ${\text{WG}}$) and backfat thickness gain (${\text{FI}}$ / ${\text{FG}}$); r_g_(x, y) = genetic correlation between components of feed efficiency

^c^Pr| $\hat{p}$ | > 0 is only relevant for the genetic correlations, prior assumptions of the heritabilities force this quantity to be equal to 1

Moreover, the sum of additive genetic plus permanent environmental variances for ${\text{RFI}}$ dropped from 33,531 (2576) (g/d)^2^ in Model 1 to 21,010 (2057) in Model 8. This suggests that animal-specific variation in feed allocation for maintenance, growth and backfat deposition jointly explain 37% of the individual variance for ${\text{FI}}$ in Model 8. The ratios of genetic variance to the total individual variation (sum of additive genetic and permanent environmental variances) were equal to 0.44, 0.39 and 0.55, for ${\text{MW}}$, ${\text{WG and }}$ ${\text{FG}}$, respectively.

Regarding genetic correlations between ${\text{FE}}$ components, the results in Table 5 indicate a positive association between feed required for overall growth (${\text{a}}_{{{\text{FI}}/{\text{WG}}}}$) and for fat growth (${\text{a}}_{{{\text{FI}}/{\text{FG}}}}$). Conversely, genetic correlations of the former variables with ${\text{FI}}$ per unit of ${\text{MW}}$ (${\text{a}}_{{{\text{FI}}/{\text{MW}}}}$) were negative; however, the probabilities that these correlations are higher or lower than 0 were not extreme, < 0.10 or > 0.90, thus their signs should be interpreted with caution. Regarding the genetic component of ${\text{RFI}}$ (the intercept), genetic correlation estimates indicated that animals with low ${\text{RFI}}$ (i.e. with high unspecific efficiency) were less efficient in the use of feed for maintenance (negative correlation with ${\text{a}}_{{{\text{FI}}/{\text{MW}}}}$), but were more efficient regarding the amount of feed required per unit of ${\text{WG}}$ or ${\text{FG}}$ (${\text{a}}_{{{\text{FI}}/{\text{WG}}}}$ and ${\text{a}}_{{{\text{FI}}/{\text{FG}}}}$).

To quantify how much each component of ${\text{FI}}$ was accounted for, heritabilities across explanatory variables were computed at − 0.75, − 0.5, + 0.5, + 0.75 standard deviations from the mean (Table 6). Statistics of the marginal posterior distributions of differences in heritability and of the genetic and phenotypic variances at − 0.75 and at + 0.75 standard deviations of the explanatory variables from the mean are also provided. When genetic parameters across a given component were considered, the other two explanatory variables were kept constant at their mean, i.e. 0.

**Table 6:** Statistics^a^ of marginal posterior distributions for heritability estimates at − 0.75, − 0.5, + 0.5 and + 0.75 sd of the explanatory covariates, and of differences in estimates of heritabilities ($\varvec{h}^{2}$) and of genetic ($\varvec{\sigma}_{\varvec{a}}^{2}$) and phenotypic ($\varvec{\sigma}_{\varvec{P}}^{2}$) variances at − 0.75 and + 0.75 sd of the explanatory covariates^b^

	$h_{@ - 0.75}^{2}$	$h_{@ - 0.5}^{2}$	$h_{@ + 0.55}^{2}$	$h_{@ + 0.75}^{2}$	Estimated differences between parameters at − 0.75 and + 0.75 sd units		
					$h^{2}$	$\sigma_{a}^{2}$	$\sigma_{P}^{2}$
$${\text{MW}}$$							
Mean	0.20	0.17	0.10	0.10	0.10	9001	− 2383
sd	0.04	0.04	0.05	0.05	0.06	6077	4278
HPD	0.12; 0.27	0.10; 0.24	0.01; 0.19	0.01; 0.19	− 0.02;0.20	− 3243;19,668	− 10,965;5913
Pr| $\hat{p}$ |>0^c^	1.00	1.00	1.00	1.00	0.94	0.92	0.29
$${\text{WG}}$$							
Mean	0.08	0.09	0.17	0.19	− 0.11	− 9600	4076
Sd	0.04	0.04	0.04	0.04	0.04	3492	2960
HPD	0.01;0.15	0.01;0.16	0.09;0.24	0.11;0.27	− 0.18;− 0.04	− 16,662;− 3027	− 1620;9978
Pr| $\hat{p}$ |>0^c^	1.00	1.00	1.00	1.00	0.01	0.02	0.92
$${\text{FG}}$$							
Mean	0.10	0.11	0.16	0.18	− 0.08	− 6690	2375
Sd	0.05	0.05	0.04	0.04	0.04	3832	2606
HPD	0.01;0.18	0.02;0.18	0.08;0.23	0.10;0.25	− 0.15;0.00	− 13,959;581	− 2763;7462
Pr| $\hat{p}$ |>0^c^	1.00	1.00	1.00	1.00	0.02	0.04	0.82

${\text{MW}}$ = mean metabolic weight; ${\text{WG}}$ = weight gain; ${\text{FG}}$ = back fat thickness gain

^a^Mean, standard deviation, highest posterior density (HPD) intervals, probability of the parameter to be higher than zero (Pr| $\hat{p}$ | > 0)

^b^For each covariate assessed along its range, the other ones were kept at their average, i.e. at zero

^c^ Pr| $\hat{p}$ | > 0 is only relevant for the differences between parameters, prior assumptions of the heritabilities force this quantity to be equal to 1

Because the estimates of the genetic correlation between intercept and slope on ${\text{MW}}$ and between intercept and slope on ${\text{WG}}$ and ${\text{FG}}$ had different signs, heritability tended to decrease as ${\text{MW}}$ increased, whereas this trend was positive for both ${\text{WG}}$ and ${\text{BF}}$. Differences between heritability estimates at − 0.75 and at + 0.75 were 0.10, − 0.11 and − 0.08 for ${\text{MW}}$, ${\text{WG}}, {\text{and}}$ ${\text{FG}}$ (Table 6), respectively. Differences between genetic variances at − 0.75 and at + 0.75 were 9001, − 9600 and − 6690, all with extremely high probabilities (> 0.9) of being larger or smaller than zero. Differences between phenotypic variances at − 0.75 and at + 0.75 sd units were smaller and had an opposite sign than the differences between genetic variances. Thus, our conclusion is that changes in heritability estimates along the axis of explanatory covariates were the consequence of changes in genetic variances. Having an opposite sign between differences in genetic and in phenotypic variances across explanatory variables indicates that correlations between genetic effects and between permanent environmental effects had opposite signs for the most relevant components of the model. For example, the genetic correlations (Table 5) between the intercept and the slopes on ${\text{MW}}$, ${\text{WG}}$ and ${\text{FG}}$ were − 0.46 (0.30), 0.75 (0.26) and 0.52 (0.31), whereas the correlations between permanent environmental effects for these slopes were 0.48 (0.20), − 0.79 (0.15) and − 0.74 (0.189) (results not shown).

### Expected responses to selection

Table 7 presents results of a simulated selection process that was conducted with different selection criteria. Selection intensity and the proportion of males and females selected in each scenario were relatively constant. Selection accuracy ranged from 0.44 to 0.61 for the different criteria, which indicates differences in genetic prediction quality. These differences, jointly with the differences in the values of the relevant genetic parameters (variances, heritabilities and correlations) for each selection criterion, result in the variation in selection responses reported in Table 8.

**Table 7:** Average (standard deviation) across 50 replicates for selection intensity, proportion of male (M) and female (F) candidates selected, and accuracy for the evaluated scenarios

Scenario	Intensity	Proportion F	Proportion M	Accuracy
${\hat{\mathbf{I}}}_{\text{FI}}$	− 1.53 (0.10)	0.15 (0.02)	0.64 (0.13)	
${\hat{\mathbf{I}}}_{\text{tRFI}}$	− 1.52 (0.09)	0.15 (0.02)	0.66 (0.13)	0.56 (0.08)
${\hat{\mathbf{I}}}_{\text{RFI}}$	− 1.53 (0.09)	0.15 (0.02)	0.61 (0.13)	0.61 (0.05)
${\hat{\mathbf{I}}}_{{{\text{FI}}/{\text{MW}}}}$	− 1.50 (0.1)	0.15 (0.02)	0.72 (0.12)	0.47 (0.09)
${\hat{\mathbf{I}}}_{{{\text{FI}}/{\text{WG}}}}$	− 1.49 (0.1)	0.15 (0.02)	0.72 (0.14)	0.53 (0.05)
${\hat{\mathbf{I}}}_{{{\text{FI}}/{\text{FG}}}}$	− 1.52 (0.09)	0.15 (0.02)	0.65 (0.14)	0.44 (0.09)

${\hat{\mathbf{I}}}_{\text{RFI}}$ = selection against the consumption of feed beyond individual needs defined by the overall needs; ${\hat{\mathbf{I}}}_{{{\text{FI}}/{\text{MW}}}}$, ${\hat{\mathbf{I}}}_{{{\text{FI}}/{\text{WG}}}}$, ${\hat{\mathbf{I}}}_{{{\text{FI}}/{\text{FG}}}}$ = selection to reduce the amount of feed consumed for maintenance, growth and fat deposition, respectively

${\hat{\mathbf{I}}}_{\text{FI}}$ = selection against feed intake; ${\hat{\mathbf{I}}}_{\text{tRFI}}$ = selection for reduced traditional ${\text{RFI}}$

**Table 8:** Average (standard deviation) across 50 replicates of responses to selection for different indexes

Response for	Index					
	${\hat{\mathbf{I}}}_{\text{FI}}$	${\hat{\mathbf{I}}}_{\text{tRFI}}$	${\hat{\mathbf{I}}}_{\text{RFI}}$	${\hat{\mathbf{I}}}_{{{\text{FI}}/{\text{MW}}}}$	${\hat{\mathbf{I}}}_{{{\text{FI}}/{\text{WG}}}}$	${\hat{\mathbf{I}}}_{{{\text{FI}}/{\text{FG}}}}$
${\text{MW}}$ (g)	− 0.29 (0.09)	− 0.02 (0.07)	− 0.04 (0.05)	− 0.07 (0.06)	0.01 (0.04)	0 (0.04)
${\text{WG}}$ (g/d)	− 0.03 (0.02)	0 (0.02)	0 (0.01)	0 (0.01)	0.01 (0.02)	0 (0.01)
${\text{FG}}$ (mm)	− 0.01 (0.01)	0 (0.01)	0 (0.01)	0 (0.01)	0 (0.01)	0 (0.01)
${\text{RFI}}$ (g/d)	− 63.18 (17.32)	− 87.03 (13.23)	− 98.62 (9.87)	45.16 (31.09)	− 43.63 (18.76)	− 21.12 (23.74)
${\text{FI}}$ /sd MW ((g/d)/g)	17.77 (9.94)	10.59 (17.52)	13.41 (8.49)	− 33.45 (6.21)	− 5.57 (6.76)	− 6.39 (7.75)
${\text{FI}}$ /sd WG ((g/d)/g)	− 2.55 (6.15)	− 10.34 (6.99)	− 15.61 (5.27)	− 4.71 (7.18)	− 34.87 (3.81)	− 29.22 (5.01)
${\text{FI}}$ /sd FG ((g/d)/g)	0.54 (2.09)	− 1.28 (3.01)	− 3.16 (2.36)	− 2.25 (2.6)	− 12 (2.11)	− 12.02 (1.8)
${\text{FI}}$ (g/day)	− 165.61 (35.14)	− 111.08 (35.83)	− 113.83 (16.07)	42.73 (49.27)	− 43.32 (28.8)	− 23.41 (34.87)

${\text{MW}}$ = mean metabolic weight; ${\text{WG}}$ = weight gain; ${\text{FG}}$ = backfat thickness gain; ${\text{RFI}}$ = residual feed intake (Model 8); ${\text{FI}}$ = feed intake; ${\hat{\mathbf{I}}}_{\text{FI}}$ = selection against feed intake; ${\hat{\mathbf{I}}}_{\text{tRFI}}$ = selection for reduced traditional ${\text{RFI}}$; ${\hat{\mathbf{I}}}_{\text{RFI}}$ = selection against the consumption of feed beyond individual needs defined by index of overall needs (Model 8); ${\hat{\mathbf{I}}}_{{{\text{FI}}/{\text{MW}}}}$, ${\hat{\mathbf{I}}}_{{{\text{FI}}/{\text{WG}}}}$, ${\hat{\mathbf{I}}}_{{{\text{FI}}/{\text{FG}}}}$ = selection to reduce the amount of feed consumed for maintenance, growth and fat deposition, respectively

Selection for reducing total ${\text{FI}}$ yielded reduced daily ${\text{FI}}$ by 166 g per generation (5.5% of mean ${\text{FI}}$ in generation 0) and a correlated negative response in ${\text{MW}}$ (− 0.29 sd units) but no responses in ${\text{WG}}$ and ${\text{FG}}$. Considering the phenotypic standard deviations in Table 2, the response for ${\text{MW}}$ translated into a decrease of 667 g per generation, which represents 2.5% of the average of this trait. Another relevant correlated response in this scenario is an unfavourable response on the efficiency for maintenance, 17.77 g of FI per sd unit of MW, although as animals eat less they become less efficient for maintaining their body structure.

Selection to reduce the traditional definition of ${\text{RFI}}$ (t ${\hat{\mathbf{I}}}_{\text{tRFI}}$) reduced ${\text{FI}}$ per day by 111 g per generation, which corresponds to 3.7% of the phenotypic mean in the base generation. When the selection criterion was EBV for the intercept in Model 8 (${\hat{\mathbf{I}}}_{\text{RFI}}$), which is the same model as used for generating the data, responses in ${\text{FI}}$ per generation were 2% higher (− 114 g/d) than for the traditional definition of RFI. According to the previous DIC results, Model 8 is the most appropriate to fit our data, thus we concluded that in our population, selecting candidates on the basis of EBV for ${\text{RFI}}$ based on a model that considers animal-specific requirements for biological functions could improve the rate of genetic gain compared to EBV predictions based on a traditional ${\text{RFI}}$ model. In both scenarios (${\hat{\mathbf{I}}}_{\text{tRFI}}$ and ${\hat{\mathbf{I}}}_{\text{RFI}}$), correlated responses in ${\text{MW}}$, ${\text{WG and }}$ ${\text{FG}}$ were null, which is consistent with the assumed zero genetic correlation of these traits with ${\text{RFI}}$. However, favourable responses were found in feed required per sd unit of ${\text{WG}}$ (− 10.34 and − 15.61 for ${\hat{\mathbf{I}}}_{\text{tRFI}}$ and ${\hat{\mathbf{I}}}_{\text{RFI}}$, respectively) and ${\text{FG}}$ (− 1.28 and − 3.16 for ${\hat{\mathbf{I}}}_{\text{tRFI}}$ and ${\hat{\mathbf{I}}}_{\text{RFI}}$, respectively), but unfavourable responses were found in feed required per sd unit of MW (10.59 and 13.41 for ${\hat{\mathbf{I}}}_{\text{tRFI}}$ and ${\hat{\mathbf{I}}}_{\text{RFI}}$, respectively).

When animals were selected for reduced ${\text{FI}}$ required per unit of ${\text{WG}}$ (${\hat{\mathbf{I}}}_{{{\text{FI}}/{\text{WG}}}}$), an important decrease in this trait was obtained as a direct response (− 34.87 gr/sd unit of ${\text{MW}}$). However, the reduction in daily ${\text{FI}}$ per generation was less than half of that achieved when considering either ${\hat{\mathbf{I}}}_{\text{tRFI}}$ or ${\hat{\mathbf{I}}}_{\text{RFI}}$. The correlated responses on efficiency for body maintenance and for fat deposition were both favourable. When the selection criterion was ${\text{FI}}$ required per unit of ${\text{FG}}$ (${\hat{\mathbf{I}}}_{{{\text{FI}}/{\text{FG}}}}$), similar responses on ${\text{MW}}$, ${\text{FG}}$ and ${\text{WG}}$ efficiencies were observed, but in this case the correlated response on ${\text{FI}}$ per generation was divided approximately by two.

Selection for efficiency on maintenance (${\hat{\mathbf{I}}}_{{{\text{FI}}/{\text{MW}}}}$) led to an important (− 33.45 g/sd unit of ${\text{MW}}$) direct response as well as to favourable responses on the efficiency for using feed for ${\text{WG}}$ and ${\text{FG}}$. However, the overall feed efficiency of the animals after this selection strategy would be reduced, since both ${\text{FI}}$ and ${\text{RFI}}$ were increased.

## Discussion

A number of difficulties appear when ${\text{FE}}$ is considered in breeding programs. The first one is associated with the effort needed to properly record feed intake in an environment similar to pig production farms, i.e. with animals raised in groups. Fortunately, the development of commercial automatic feeders can overcome this issue. The second difficulty is associated with the choice of the selection criteria. Feed efficiency is by nature a composite trait, which on the one hand involves intake traits and, on the other, performance traits. For the particular case of growing pigs, performance traits usually include growth rate, backfat deposition and maintenance needs. In this sense, from an applied and commercial perspective, a multiple-trait evaluation model for these traits and ${\text{FI}}$, combining breeding value predictions based on economic weights is expected to yield optimal results with regard to the overall economic response to selection [20]. Nevertheless, in order to explore particular aspects of feed efficiency, the use of phenotypic indexes that combine feed intake and animal’s performances could be relevant. In this sense, ${\text{RFI}}$ has been proposed as an alternative selection criterion and has been used in several selection experiments [6, 7], yielding favourable responses in ${\text{FE}}$. The main reason for claiming ${\text{RFI}}$ as a good alternative for improving ${\text{FE}}$ is its null phenotypic correlation with traits that define the animals’ needs, i.e. backfat deposition, growth rate and maintenance needs.

Two major negative points are associated with the use of ${\text{RFI}}$ as a selection criterion. On the one hand, as a consequence of the traditional definition of ${\text{RFI}}$, based on a multiple fixed regression of ${\text{FI}}$ on metabolic weight, weight gain and backfat deposition for groups of animals, animals with poor performance can appear within the group of candidates, i.e. slow growing pigs with a low intake that would be considered as efficient and selected. If regression is nested within individuals, the corrections of ${\text{FI}}$ are done for individual needs and the chances of selecting animals with a poor performance (with respect to the group) as efficient animals are reduced [10].

On the other hand, it has been claimed that selection to reduce ${\text{RFI}}$, without distinguishing between different biological functions, might have negative consequences on some of these biological functions. A classical example would be that animals selected for ${\text{RFI}}$ might reduce the amount of energy used for maintaining physiological functions and for coping with challenging situations, such as the occurrence of disease or poor diets. Fitting ${\text{FI}}$ by using models that include animal-specific nested regressions on ${\text{MW}}$, ${\text{WG}}, {\text{and }}$ ${\text{FG}}$ at the genetic level can be used to genetically alter the efficiency associated with one function but not another, provided they are not highly correlated. In addition, these models could be used to study the biological basis and the genes involved in each of the biological functions [22].

### Genetic parameters

Recently, Martinsen et al. [11] considered a similar approach to study the efficiency to deposit fat and lean meat in two Norwegian pig breeds. Their objective was to estimate variance components of the proposed model, without testing the relevance of each component. They observed that a full model that jointly accounted for animal-specific variation in feed requirements per unit of metabolic weight, lean growth, and backfat thickness did not converge properly. Our Gibbs sampler chains converged in all the models studied, even in the most complete model. However, the chain quality dropped as the complexity of the model increased. Martinsen et al. [11] used explanatory variables that were quite different to those in our study. In addition, they did not fit regressions on metabolic body weight, resulting in their findings to be not fully comparable with ours. Nevertheless, it should be noted that the sign of their genetic correlations estimates of the intercept with feed requirements for lean meat growth and fat growth coincide with those that we obtained between intercept and feed requirements for overall growth and backfat deposition. However, Martinsen et al. [11] reported a nearly null correlation between feed requirements for lean meat and fat content, while we observed a clearly favourable correlation between feed requirements for overall growth and backfat deposition. These differences could be explained by the negative correlations that we observed between the use of feed for maintenance and the other ${\text{FE}}$ components (${\text{RFI}}$ and the use of feed for growth and fat deposition).

Our estimates of genetic correlations that involve ${\text{RFI}}$ (Table 5) mean that animals that have favourable genetic effects for efficiency with respect to unspecific factors (intercepts) will require more feed to maintain an extra unit of metabolic weight. In other studies that fitted similar models [9, 10] random intercepts were not considered, or at least not their genetic component. Thus, these results are also not directly comparable to ours.

Previous experimental results have shown that the positive response that is achieved for efficiency based on selection for reduced ${\text{RFI}}$ is a consequence of both better use of feed to cope with maintenance needs [23, 24] and a reduction of other functions that influence efficiency, for example physical activity [23, 25]. However, none of these two experiments reported a better efficiency in protein or fat deposition after selection. For these results to be fully explained by our complete model (Model 8), the genetic correlation between intercepts and feed per unit of ${\text{MW}}$ would have to be positive, while those between intercepts and feed per unit of ${\text{WG}}$ and ${\text{FG}}$ would have to be null, similar to those between feed per unit of ${\text{MW}}$ and feed per unit of ${\text{WG}}$ and ${\text{FG}}$. However, we obtained different genetic correlations in our Duroc population but it must be noted that the genetic origins of the Duroc line and of those used in the aforementioned experiments are completely different and thus differences in the genetic control of ${\text{FE}}$ components can be expected.

To our knowledge, there is no literature on the further biological assessment of selection to improve ${\text{FE}}$ in pigs. Similar results have been reported in layers [26] and beef cattle [27]. However, another study in beef cattle did not detect differences in maintenance needs between lines with divergent ${\text{RFI}}$ [28]. Other factors that may explain differences in efficiency resulting from selection for ${\text{RFI}}$ are related to digestibility [29]. In our study, this factor was not explicitly fitted but its effect could be considered through the random intercept in the model. Unfortunately, the lack of experimental results on our population did not allow us to carry out a direct biological validation of the proposed model.

### Expected responses to selection

To complete the discussion on expected responses in the components of ${\text{FE}}$ after selection for ${\text{RFI}}$, both the proportion of individual ${\text{FI}}$ variability that is explained by each component and their heritabilities should be considered, in addition to the genetic correlations previously discussed. To implicitly account for all these parameters, we conducted several simulation tests. Although the data were generated using Model 8, selection for traditional ${\text{RFI}}$ (${\hat{\mathbf{I}}}_{\text{tRFI}}$) yielded similar results (in terms of reductions in ${\text{FI}}$) as selection for reduced EBV for the intercept from Model 8 (${\hat{\mathbf{I}}}_{\text{RFI}}$). These similar results are due to the fact that random intercepts, which correspond to unspecific use of feed, account for most of the variation in ${\text{FI}}$ (63%). With regard to correlated responses in ${\text{FI}}$ components, a reduction in the amount of feed used for growth was found when selection was either on ${\hat{\mathbf{I}}}_{\text{RFI}}$ or on ${\hat{\mathbf{I}}}_{\text{tRFI}} .$ These correlated responses are implicitly considered in the response for total ${\text{FI}}$ (− 113.83 and − 111.08). However, if the selection index, also included EBV for growth, in addition to EBV for ${\text{RFI}}$, ${\text{FI}}$ would be further reduced per each g of improved daily growth, i.e. the progress in growth will be achieved at lower feed cost. Taking advantage of this extra reduction in ${\text{FI}}$ would, of course, be possible only if the genetic correlations between ${\text{RFI}}$, ${\text{FI}}$ per unit of growth, and growth are favourable. Note however that by this procedure the animals would become less efficient in maintaining body structure, thus if selection for growth increases the metabolic weight of the animals, part of the aforementioned extra gain in feed efficiency would be counterbalanced by the unfavourable response on efficiency for maintenance.

When selection aims at reducing the amount of feed devoted to growth (${\hat{\mathbf{I}}}_{{{\text{FI}}/{\text{WG}}}}$) or backfat deposition (${\hat{\mathbf{I}}}_{{{\text{FI}}/{\text{FG}}}}$), successful direct responses were achieved, and correlated responses on overall ${\text{FI}}$ were also obtained, but in this case the magnitude of this response would be less than half that achieved when the selection criteria are either ${\hat{\mathbf{I}}}_{\text{RFI}}$ or ${\hat{\mathbf{I}}}_{\text{tRFI}} .$ This correlated response became unfavourable when the selection criterion was feed devoted to maintenance (${\hat{\mathbf{I}}}_{{{\text{FI}}/{\text{MW}}}} ).$ Thus, when the objective is to improve overall feed efficiency, the focus should be on random intercepts (residual intake).

Responses in terms of reductions in ${\text{FI}}$ in real selection experiments for RFI were much lower than those found in our study. For example, Sellier et al. [30] reported a reduction in ${\text{FI}}$ of 19 g per generation after selection for traditional ${\text{RFI}}$. Our responses were 5 to 6 times higher, but there are major differences with regard to the number of animals with records and selection intensities between our simulation design and those applied in real selection trials. In spite of these differences, the simulation study is useful for comparing alternate selection criteria.

The proposed models have limitations that need to be considered. These points are relevant for both the traditional ${\text{RFI}}$ definition and for the new definitions that we explored here. One of these points is that genetic correlations of the independent traits, i.e. performance traits reflecting animal’s needs, with the components of the trait of interest (random intercepts or regressions) are assumed to be zero. This was previously addressed in the framework of traditional ${\text{RFI}}$ [21]. The consequences of not considering such correlations are that selection for ${\text{RFI}}$ can lead to correlated responses in the explanatory traits (back fat thickness or growth) [7]. Structural equation models (SEM; [31]) could offer the framework to account for these correlations. Nevertheless, the implementation of SEM supposes the use of multivariate models with a very large number of parameters, which will not be properly estimated when the number of records is limited.

The other point to consider is how well the explanatory variables reflect the intended biological functions. This is particularly true for metabolic weight as a trait that accounts for maintenance needs. Given the information that is available in most breeding programs, it is not possible to propose alternative traits to reflect maintenance needs. Thus, an important activity for the future will be to explore alternative easy-to-measure traits that better reflect the maintenance needs of animals. In the work by Martinsen et al. [11] computed tomography records were used to properly assess body composition. These computed tomography records could be highly relevant to explore the role of these records as predictors of experimentally-recorded maintenance needs.

The final point that needs to be considered is that Model 8 involves a larger number of parameters than the traditional definition of ${\text{RFI}}$. This implies that the parameters will be estimated with lower accuracy, which may have negative consequences in the final response to selection. In our simulation test, we generated large datasets (approx. 1500 feed intake records per generation, including on all candidates, both males and females), such that model parameters could be properly estimated. Having fewer records per generation would reduce the slight superiority of Model 8 over the traditional definition of ${\text{RFI}}$.

## Conclusions

Animal-specific needs should be included in models for genetic evaluation of feed efficiency. The model that accounts for animal-specific feed requirements per unit of growth, backfat thickness gain and metabolic body weight needs was statistically superior to the traditional ${\text{RFI}}$ model, which considers population-specific needs for these components. However, response in overall efficiency was only slightly greater when a model with animal-specific needs was used, which is because individual variation in the components of feed efficiency represents only about one third of the variation in the individual’s ${\text{FI}}$. Part of this slightly higher response in feed efficiency was obtained because the improved animals were more efficient in the use of feed for growth, which could be highly valuable if the overall selection index includes EBV for growth, in addition to EBV for ${\text{RFI}}$. Selecting animals for the efficiency associated with specific biological functions would be possible with the proposed model fitting the genetic components of animal-specific needs. Nevertheless, genetic parameters should be properly estimated because large estimation errors would likely reduce the effectiveness of the proposed selection criteria.
